# Investigating the prevalence of and factors associated with depressive symptoms among urban and semi-urban school adolescents in Bangladesh: a pilot study

**DOI:** 10.1093/inthealth/ihz092

**Published:** 2019-11-06

**Authors:** Afifa Anjum, Sahadat Hossain, Tajuddin Sikder, Md Elias Uddin, Dewan Abdur Rahim

**Affiliations:** 1 Department of Public Health and Informatics, Jahangirnagar University, Savar, Dhaka 1342, Bangladesh; 2 Maternal and Child Health Division, International Centre for Diarrhoeal Diseases Research, Bangladesh, 68 Shahid Tajuddin Sarani, Mohakhali, Dhaka 1212, Bangladesh; 3 Department of English, University of Dhaka, Dhaka 1000, Bangladesh; 4 Department of Psychiatry, BRB Hospitals Limited, 77/A East Rajabazar, West Panthapath, Dhaka 1215, Bangladesh

**Keywords:** Bangladesh, depressive symptoms, lifestyle, screen-based sedentary behaviour, urban and semi-urban adolescents

## Abstract

**Background:**

Adolescent depression is an alarming issue for Bangladesh since a large number of Bangladeshi adolescents suffer from depression but cannot get proper treatment. This study aimed to investigate the prevalence of depression and factors associated with this psychological health hazard among urban and semi-urban adolescents of the Dhaka region in Bangladesh.

**Methods:**

A cross-sectional pilot study was performed among 311 adolescents, grades 8–10, in Dhaka city and Savar Upazila, adjacent to the city, in 2018. Data were collected using a questionnaire consisting of items on sociodemographics, lifestyle information, screen-based sedentary behaviour (SBSB) and mental health.

**Results:**

A total of 36.6% of the adolescents reported depressive symptoms, with a greater prevalence among females (42.9%) than males (25.7%). Sociodemographic factors including being female, residence and grade in school were significantly associated with depression. Logistic regression analysis showed that SBSBs, such as the use of social media (odds ratio [OR] 2.06 [95% confidence interval {CI} 1.27–3.35]), high screen time (>120 min/day; OR 2.35 [95% CI 1.30–4.25]) and sleep disturbance (OR 3.93 [95% CI 2.37–6.50]) were significantly linked with depressive symptoms.

**Conclusions:**

Depressive symptoms are prevalent among urban and semi-urban adolescents of Dhaka, Bangladesh. Therefore, urgent initiatives should be taken to curb the spread of depression among Bangladeshi adolescents.

## Introduction

Adolescence is the period between 10 and 19 y of age when individuals transition from childhood to adulthood. During this period, substantial physical, psychological and behavioural changes occur in boys and girls.^1^ There is agreement about the considerable differences between children and adolescents in all societies, although the definition and recognition of childhood and adolescence may vary across societies.^2^ According to an editorial in Nature, 25% of the total global population now belong to the 10–24 y age group, and addressing their needs is a crucial task.^3^ A WHO report from 2018 stated that 1.2 million adolescents died around the world, mostly from preventable or treatable diseases or causes.^4^

Depression, a major contributor to the global burden of diseases, is also one of the leading causes of illness and disability among adolescents.^5^ It is a great public health concern that the onset of depressive disorders is reported to occur during the period of adolescence.^6,7^ Evidence has shown that early life depression is associated with poor psychosocial outcomes, including lower social support and lower educational attainment.^8^ A systematic review of longitudinal studies showed that adolescents suffering from depression are more likely to experience at least one recurrent episode of depression in adulthood.^8^ Thus adolescent depression is a crucial public health concern because of its high prevalence and recurrence, as it causes greater risks of suicide and other comorbid psychiatric disorders and contributes to the overall disease burden.^9^ However, adolescent depressive disorders do not receive adequate attention in most cases, leading to the recurrence of depression in later adult life.^7^ Predictors of depressive disorders among adolescents include genetic and social factors, lifestyle factors, stress, anxiety and attention deficit hyperactivity disorder.^7,9–11^ Physical inactivity and screen-based sedentary behaviour (SBSB) have increased significantly among adolescents, in both developed and developing countries, which is leading them towards depressive symptoms.^10,11^ Sleeplessness and sleep disturbance have been found to be associated with depressed mood among adolescents.^12,13^

Data regarding adolescent depression are mostly available for high-income industrialized countries, although evidence is available for low-resource areas as well. In the USA, among adolescents 13–18 y of age, 14% met the criteria for mood disorder^14^ and 12.5% suffered from depressive disorder. One in nine adolescents is affected by depression in a given year, and an adolescent facing depressive symptoms suffers from subsequent episodes in adulthood.^15^ Balasz et al.^16^ found that 10% of 13 000 young Europeans showed significant depressive symptoms. In the BELLA study in Germany, it was found that in a random sample of 3256 consisting of youths aged 7–19 y, 11.2% were experiencing depressive symptoms; moreover, self-reports from the same respondents showed that 16.1% suffered from depressive symptoms.^17^ Dardas et al.^18^ found that among 2349 Jordanian adolescents, the mean total depression score was 16.3 and 34% of them reported moderate to severe depression. Depression among adolescents in low- and middle-income countries (LMICs) is not uncommon and it causes great harm to their minds and bodies. It is highly prevalent among adolescents in Southeast Asia, where adolescents comprise one-tenth of the regional inhabitants.^1^ A study in Pakistan showed that 72% of adolescent girls suffered from some level of depression.^19^ In India, the prevalence of depression among adolescents was 22.45%, and girls were found to be more vulnerable than boys.^20^ A study conducted on 893 adolescents in two districts of Nepal found that the prevalence of depression was 40.4% in Sindhupalchok and 23.2% in Kathmandu.^21^ A study in Sri Lanka found that 17% of adolescents were suffering from mild depression and 19% from severe depression.^22^

In Bangladesh, adolescents comprise 10.2% of the total population (16.4 million; 8.4 million boys and 8.0 million girls). Studies have shown that adolescents of Bangladesh suffer greatly from anxiety, loneliness, lack of close friends, bullying, substance abuse and smoking.^1^ Although a large proportion of students suffer from these problems, this issue has remained little researched, with only one study conducted on the rural and underprivileged adolescents in Bangladesh^23^ and another on adolescents of Dhaka city.^10^ However, no studies have yet been conducted on adolescents in urban and semi-urban settings in Bangladesh. Therefore, the purpose of this study was to identify the prevalence of depression and the factors associated with this psychological health hazard among urban and semi-urban adolescents in the Dhaka region of Bangladesh. We hypothesized that a school residential setting would be a predictor of depressive symptoms, and urban adolescents would have higher risks of depression than their semi-urban counterparts. We also assumed that demographic variables such as gender, age, student grade and family would be associated with this psychological health problem. Furthermore, students’ lifestyle-related variables would be significant predictors of adolescent depression. Therefore, in line with prior research on physical inactivity,^11^ high SBSB,^24,25^ sleep disturbance^12,13^ and depressive symptoms, we assumed these lifestyle variables to be the risk factors for depressive symptoms among Bangladeshi adolescents.

## Materials and methods

### Study design and setting

This cross-sectional pilot study was conducted in two different areas—one urban and the other semi-urban—between 15 July 2018 and 20 September 2018. In this study, Dhaka city was the urban area where Dhanmondi thana was selected as the study site, and Savar Upazila, adjacent to the city of Dhaka, was the semi-urban area where Savar thana was selected as the study site. These two thanas were selected for this pilot study, considering the intra-thana homogeneity of the population.

### Study procedures

We initially listed all the secondary schools of the two thanas and found that there were 16 schools in Dhanmondi thana and 14 schools in Savar thana. However, two schools were selected considering the diverse socio-economic background of the participants and their accessibility to the research team. In Bangladesh, grades 6–10 fall under secondary school education. As such, adolescents in grades 8–10 (ages 13–17 y) constituted the population of this study. The research team then collected a list of enrolled students from the school authorities. A probability sampling technique was used to determine the sample size of this study, which was 347, with a 20% non-response rate. With the permission of the headmasters/principals of the schools and then class teachers, the research team went to the classrooms and explained the rationale and purpose of the study to the students. The students were also notified that they would have to obtain permission from their parents to participate in the study. After the research team had obtained confirmation of individual informed consent from the students and their parents, 311 students completed the survey questionnaire (90% response rate) in the classroom under the supervision of the lead researcher. A teacher and a member of the research team were also present to monitor the progress of the survey and to answer any questions or address any concerns of the participants. Non-consenting students engaged in alternative activities of their choice during this time. We believe that the respondents of this study represent the entire population of adolescents in the urban and semi-urban areas in the study, as the urban areas in Bangladesh have similar demographic and socio-economic conditions, and the same is true for the semi-urban regions of the country.

### Outcome measures

In order to measure the level of depression of the participants, this study used the nine-item Patient Health Questionnaire (PHQ-9), corresponding to the Diagnostic and Statistical Manual of Mental Disorders, Fourth Edition diagnostic criteria of symptoms for major depressive disorder. The PHQ-9 possesses good sensitivity (88%) and specificity (94%) for measuring the severity of depression among both the clinical and general population samples.^26^ Study findings show that the PHQ-9 also had high sensitivity (89.5%) and good specificity (78.8%) for detecting major depression among adolescents, and it has almost similar sensitivity and specificity for detecting depressive symptoms among adults.^27^ Also, the sensitivity and specificity of this tool are quite similar to those of other depression screening tools tested among adolescents in primary care: Beck Depression Inventory (sensitivity 91%, specificity 91%),^28^ PHQ-9 modified for adolescents (sensitivity 73%, specificity 94%)^29^ and the Short Mood and Feelings Questionnaire (sensitivity 80%, specificity 81%).^30^ Thus the PHQ-9 turns out to be a promising screening tool for use among adolescents. In our study, we also found that the PHQ-9 is a highly reliable (Cronbach’s α = 0.83 [95% confidence interval {CI} of intraclass correlation coefficient 0.80–0.86]) scale for Bangladeshi adolescents.

Participants were asked how often they were bothered by each of the depressive symptoms, with four response options: 0 = not at all, 1 = several days, 2 = more than half the days and 3 = nearly every day over the last 14 days. The response options were also treated as a continuous ordinal measure. Therefore, the range of scores for the PHQ-9 was 0–27. The cut-off points for the categorization of depressive symptoms were as follows: 0–4 = normal, where no depression symptoms appeared, hence no depression treatment was required; 5–9 = mild depressive symptoms, where psychological support and educational counselling were required; 10–14 = moderate depressive symptoms, where clinical judgement about treatment was needed based on the subject’s duration of symptoms; 15–19 = moderately severe depressive symptoms, where a combination of treatment with drugs and therapy was needed; and ≥20 = severely severe depressive symptoms, where urgent specialized treatment with drugs and therapy was required.

### Other measures

#### Sociodemographics

Sociodemographic data collected from the participants included their age, gender, grade in school, residence, birth order, religion, parents’ educational status and monthly family income.

#### Lifestyle

To determine the lifestyle of the adolescents, this study observed three factors: physical activity (PA), SBSB and sleeping status. The questionnaire included response options on three different levels of PA: low-level PA (unintentional walking <30 min/d), moderate PA (walking or meditation/yoga ≥30 min/d) and vigorous PA (jogging, cycling, playing sports or gym workouts ≥60 min/d). In the case of inactivity, the respondents were asked how long they had been inactive. For SBSB, participants were asked whether they watched live movies/plays or YouTube videos and spent time on the internet using social media such as Facebook and Twitter. Patterns of watching live movies/plays or YouTube videos and internet use were categorized based on hours per day, week and month. High recreational screen time was defined as >2 h/d, which is consistent with a widely used screen time recommendation.^24,31^

### Statistical analyses

Data analysis involved descriptive statistics as well as inferential statistics. For the identification of significant relationships between the study variables, test statistics such as the χ^2^ test were used. Logistic regression models were used when it was assumed that variables had an association with outcome variables. During regression analysis, data were adjusted for various factors and were reported for the adjusted odds ratios (ORs) with 95% CIs. The level of significance was set at p<0.05. All data were analysed using the Statistical Package for the Social Sciences software for Windows, version 22.0 (IBM, Armonk, NY, USA).

## Results

**Figure 1:**
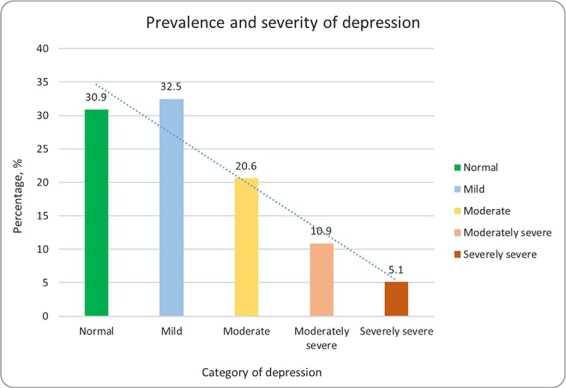
Prevalence and severity of depression among adolescents in Dhaka, Bangladesh.

Of 311 participants, a majority were females (63.7% [n=198]) and class 8 students (52.7% [n=164]). The participants’ ages ranged from 13 to 17 y, with a mean of 14.63±0.99 y. Four in ten participants (44.1%) reported that they were the first child of their parents. For the residential setting, 53.3% hailed from a semi-urban setting and 44.7% were from an urban setting. The prevalence and severity of depression among the participants are shown in Figure 1. We found that 5.1% of the participants suffered from severely severe depression, while 30.9% were in a normal state of depression, 32.5% of the participants were found to experience mild depression, 20.6% were found to be experiencing moderate depression and 10.9% were experiencing moderately severe depression at the time of data collection.

This study demonstrated that females (42.9%) suffered more from depression than males (25.7%) (Table 1), which was highly statistically significant (p=0.002). The highest percentage (47.5%) of students suffering from depression were from class 9, and this was also highly statistically significant (p=0.004). Furthermore, 47.5% of the participants in the urban setting were found to be depressed, whereas 42.1% of the participants in the semi-urban setting were found to be depressed. According to birth order, participants whose position was third or more were found to be the most depressed (48.6%) compared with the second (32.7%) and first (33.6%) positions.

**Table 1:** Association of sociodemographic variables with depression

Variables	Depression^a^	
	Frequency (% within variable)	χ^2^ value (p-value)
Gender		
Male	29 (25.7)	9.24 (0.002)**
Female	85 (42.9)	
Student grade		
Class 8	46 (28.0)	11.14 (0.004)**
Class 9	29 (47.5)	
Class 10	39 (45.3)	
Age (years)		
13	11 (29.7)	8.62 (0.087)
14	32 (28.1)	
15	41 (44.1)	
16	27 (45.0)	
17	3 (42.9)	
Birth order		
1	46 (33.6)	5.54 (0.063)
2	34 (32.7)	
≥3	34 (48.6)	
Father’s level of education (n=222)		
Primary	8 (23.5)	4.91 (0.086)
Secondary/higher secondary	24 (27.3)	
Graduate/above	40 (40.0)	
Mother’s level of education (n=230)		
Primary	21 (33.3)	0.95 (0.62)
Secondary/higher secondary	40 (31.0)	
Graduate/above	15 (39.5)	
Total number of family members		
≤4	54 (34.4)	0.70 (0.40)
≥5	60 (39.0)	
Residence		
Urban	66 (47.5)	12.69 (<0.001)**
Semi-urban	48 (42.1)	

a The cut-off of PHQ-9 ≥10 is used for the depressive analysis.

** The p-value is significant at p<0.01.

This study showed a significant relationship between lifestyle pattern and depression, as presented in Table 2. Among the participants involved in PA, 33.5% were found to be depressed. Conversely, 44.1% of the participants who were physically inactive were depressed. Depression was found among 38.9% of the participants who had irregular PA and among 34% of those involved in regular PA. Although there was no statistically significant difference between the duration of daily PA and depression, it was found that depression decreased with an increase in the duration of daily PA. In this study, 43.4% of the participants who reported using social media were found to suffer from depression. Moreover, 53.7% of the students with a duration of SBSB of >2 h/d were found to be depressed. Additionally, 50.6% of the respondents who reported sleep disturbances were found to experience depressive symptoms.

**Table 2:** Association of lifestyle variables with depression

Variables	Depression^a^	
	Frequency (% within variable)	χ^2^ value (p-value)
Physical activity		
Involved in physical activity		
Yes	73 (33.5)	3.15 (0.076)
No	41 (44.1)	
Regular physical activity		
Yes	49 (34.0)	0.798 (0.372)
No	65 (38.9)	
Duration of daily physical activity (n=246)		
<30 min/d	37 (33.9)	4.62 (0.202)
30–60 min/d	20 (33.3)	
>60 min/d	14 (29.8)	
Physical activity time (n=246)		
Early morning	30 (34.1)	4.01 (0.261)
Late afternoon	37 (31.9)	
Evening	5 (38.5)	
SBSB		
Use of social media (e.g. Facebook)		
Yes	79 (43.4)	8.61 (0.003)**
No	35 (27.1)	
Duration of daily SBSB		
≤2 h/d	85 (33.1)	8.18 (0.004)**
>2 h/d	29 (53.7)	
Sleep quality		
Satisfaction about daily sleep		
Yes	30 (20.7)	29.83 (<0.001)**
No	84 (50.6)	
Sleep habit		
Short sleep duration (<7 h/d)	62 (40.0)	1.54 (0.464)
Ideal sleep duration (7–9 h/d)	48 (33.1)	
Long sleep duration (>9 h/d)	4 (36.4)	

a The cut-off of PHQ-9 ≥10 is used for depressive analysis.

* The p-value is significant at p≤0.05.

** The p-value is significant at p<0.01.

Bivariate analysis showed that being female (p=0.003), studying in higher grades like class 9 (p=0.006) and class 10 (p=0.007), residing in an urban area (p=0.000), use of social media (p=0.004), spending >2 h/d on screen (p=0.005) and daily sleep dissatisfaction (p=0.000) were significantly associated with depression among school adolescents (Table 3). However, in multivariate analysis, studying in class 9 (p=0.035), not involved in PA (p=0.050), spending >2 h/d on screen (p=0.001) and sleep dissatisfaction (p=0.000) were found to be linked with depression.

**Table 3:** Association between predictive study variables and depressive symptoms among adolescents in Dhaka, Bangladesh^a^

Variables	Unadjusted estimates			Adjusted estimates^b^		
	OR	95% CI	p-Value	OR	95% CI	p-Value
Sociodemographic variables						
Gender						
Female	2.18	1.31–3.62	0.003	1.93	0.83–4.51	0.127
Male	1			1		
Student grade						
Class 10	2.13	1.24–3.67	0.007	2.22	0.75–6.54	0.148
Class 9	2.33	1.27–4.27	0.006	2.56	1.07–6.15	0.035
Class 8	1			1		
Age (years)						
17	1.77	0.34–9.27	0.498	0.91	0.12–6.93	0.926
16	1.93	0.81–4.61	0.137	1.39	0.36–5.35	0.635
15	1.86	0.83–4.21	0.134	1.24	0.42–3.62	0.701
14	0.92	0.41–2.08	0.846	1.01	0.41–2.54	0.976
13	1			1		
Residence						
Urban	2.34	1.46–3.74	0	1.05	0.47–2.34	0.898
Semi-urban	1			1		
Lifestyle variables						
Involved in physical activity						
No	1.57	0.95–2.57	0.077	1.84	0.98–3.44	0.05
Yes	1			1		
Use of social media (e.g. Facebook)						
Yes	2.06	1.27–3.35	0.004			
No	1					
Duration of daily screen time						
>2 h/d	2.35	1.30–4.25	0.005	3.24	1.60–6.59	0.001
≤2 h/d	1			1		
Satisfaction about daily sleep						
No	3.93	2.37–6.50	0	4.35	2.33–8.11	0
Yes	1			1		
Sleep habit						
Long sleep duration (>9 h/d)	1.16	0.32–4.14	0.825	1.13	0.28–4.60	0.87
Short sleep duration (<7 h/d)	1.35	0.84–2.16	0.216	0.59	0.32–1.10	0.098
Ideal sleep duration (7–9 h/d)	1			1		

a Estimates are based on binary logistic regression with depression, PHQ-9 ≥10 scores, as a dependent variable.

b Adjusted for all presented variables in the table except use of social media (e.g. Facebook).

## Discussion

Mental health is one of the vital parameters for a happy, healthy and productive life; however, mental disorders do not often receive enough attention and are not adequately addressed as a public health issue in countries like Bangladesh. Therefore, this study conclusively reveals the most updated picture of depressive symptoms among adolescents in Dhaka, Bangladesh.

The findings of the present study indicate that self-reported depressive symptoms are common among adolescents in the Dhaka district of Bangladesh, with a prevalence of 36.6%. This is much higher than the prevalence (14%) reported in a study conducted in 2012 among 2440 Bangladeshi adolescents aged 13–19 y^23^ and the prevalence (25%) reported in another study conducted in 2013 among 898 adolescents in Dhaka, Bangladesh.^10^ However, the prevalence of depressive symptoms found in the present study is lower than that reported in a study conducted in 2012 (49%) on 165 urban older adolescents aged 15–19 y (as opposed to 13–17 y in the present study) selected from two urban schools in Bangladesh.^32^ Furthermore, the present study reported a much higher OR of depression among older adolescents than among younger ones. Depressive symptoms were less common (36.6%) in the present study than in some other Asian studies: 38% among Iranian male adolescents (average age 15.44 y [SD 0.68])^33^ and 59% among Indian adolescents aged 15–18 y.^34^ These differences in prevalence might be caused by different cultures viewing depressive symptoms differently.^35^ As in the Iranian study, the predictors of adolescent depression included a low level of self-efficacy, a high level of perceived stress and academic condition, as well as demographic factors such as the father’s occupation. In the Indian study, dissatisfaction with academic achievement, working mothers and poor familial relationships were the predictors of adolescent depression. However, in the context of this study, educational attainment of the parents, their occupation and family size and bonding were not associated with adolescent depression. One possible reason for this finding might be the comparatively small sample size of the pilot study. Another important point to note here is that in Bangladesh, women usually have the primary responsibility for raising children. After marriage, most women do not engage in jobs outside of the home; instead, they concentrate on homemaking activities. Our study reported that about 86% of the mothers were homemakers. Thus the female parents’ care and attention for adolescents might compensate for the male parents’ lack of availability for their children to a considerable extent. However, more in-depth observations or large-scale studies should be conducted to explore the causal relationship between children’s psychological disorders and familial factors.

In the present study, female adolescents had more than twice the OR of depressive symptoms than their male counterparts, which is consistent with the findings of research conducted in other Asian countries.^22,23,34^ Research findings suggest that female adolescents have more challenges during this transitional period of life because of the significant changes associated with puberty, including morphological development (e.g. growth of secondary sex characteristics), physiological changes (e.g. hormonal concentrations) and other bodily manifestations (e.g. skin changes, growth spurt and menstrual period).^36^ In addition, various social taboos and gender role norms in Asian societies may predispose females to depressive symptoms. Females may experience social restrictions and pressures, constrained opportunities and gender discrimination in Asian societies.^37^ Due to the social taboo attached to sexuality, there is often a lack of meaningful dialogue between adolescents and their parents about pubertal changes and their potential impact on adolescents’ emotional and physical health, especially among females.^37^ Consistent with our expectation, this study found a significant difference in depressive symptoms between urban and semi-urban participants. With reference to the residential setting, the urban participants had a 2.34 times (95% CI 1.46–3.74) higher risk of suffering from depression than the semi-urban participants.

This study also showed a significant association between self-reported adolescent sleep disturbance and depressive symptoms, which is consistent with the findings of other research.^12,13^ Sleep disturbances were previously reported to be significantly associated with the severity of depression, the prevalence of which was highest with insomnia.^13^ Depression and sleep disturbance are likely to have a bidirectional relationship. A study among 3186 Chinese adolescents 13–18 y of age reported that those with depressive symptoms were also at a higher risk of sleep disturbance.^12^ Another cohort study found that sleep disturbance among Chinese adolescents predicted the development and persistence of depressive symptoms.^38^ The findings of the current study suggest that depressive symptoms are more common among Bangladeshi adolescents with high recreational screen time. Participants who reported >120 min/d of recreational screen time were three times more likely to report depressive symptoms than adolescents who reported recreational screen time of <30 min/d. This is particularly important for developing countries like Bangladesh, where increasing access to technology is resulting in increasing screen use and inactivity. In Bangladesh, high screen time is ubiquitous, with 80% of adolescents in Dhaka city reporting >120 min/d of screen time,^31^ and about one-third of the adolescents do not meet PA recommendations.^32^ These results may be relevant for adolescents in other developing countries. This study also found a significant association between physical inactivity and depressive symptoms. Adjusted estimates in regression analysis showed that the adolescents who reported physical inactivity had higher odds (OR 1.84) of depressive symptoms. Previous studies also reported independent associations of high screen time and physical inactivity with psychological distress^24,39^ and interaction effects of screen time and physical inactivity on poor mental health.^25^

Despite being a burning issue of the present times, depressive symptoms among adolescents have been little researched. Moreover, to the best of our knowledge, no study has yet been conducted on the adolescents of both urban and semi-urban residential settings in Bangladesh. Therefore, the findings of this study could be used in addressing adolescent health hazards as well as formulating policies in this regard. Similarly, the current study creates avenues for further studies among urban, semi-urban and rural adolescents in Bangladesh.

This study has its limitations as well. As this was a cross-sectional study, data were collected at one point in time. Therefore, the findings might not represent the scenario perfectly and, as such, should be used with caution. Further, the use of a self-reported questionnaire in this study could lead to some recall bias. Additionally, there is a need for conducting similar studies on larger samples from different schools with wider geographic areas so that the results can be generalized.

## Conclusions

Around the world a great proportion of individuals, from children to the elderly, are suffering from mental and psychotic illnesses. Various physical, psychological and social factors render adolescents more susceptible to a wide range of mental health issues, which may impinge on their later life. Although adolescent mental health is a very important issue for any country, it has not yet received any significant attention in Bangladesh. As a result, mental health illnesses are taking a heavy toll on the adolescents of the country. As the present study revealed, a good number of Bangladeshi adolescents are suffering from depression because of various personal, academic, social and familial factors. This is a public health issue of great concern and, as such, large-scale research should be carried out for gleaning adequate data that can be used in formulating national-level policies to combat adolescent health hazards in Bangladesh.

## Authors’ contributions

AA conceived the study. AA and SH were responsible for the implementation, analyses and interpretation of data and wrote the manuscript. MTS and MEU critically reviewed and edited the manuscript. DAR supervised the study. All authors read and approved the final manuscript.

## Funding

None.

## Competing interests

None declared.

## Ethical approval

International ethical guidelines for biomedical research involving human subjects were followed throughout the study. After approval of the research proposal, ethical permission for data collection was received from the Department of Public Health and Informatics, Jahangirnagar University, Savar, Bangladesh. Furthermore, all participants read, understood and signed a written consent form at the time of survey data collection.
